# Human kallikrein-related peptidase 12 stimulates endothelial cell migration by remodeling the fibronectin matrix

**DOI:** 10.1038/s41598-018-24576-9

**Published:** 2018-04-20

**Authors:** T. Kryza, C. Parent, J. Pardessus, A. Petit, J. Burlaud-Gaillard, P. Reverdiau, S. Iochmann, V. Labas, Y. Courty, N. Heuzé-Vourc’h

**Affiliations:** 1 INSERM, Centre d’Etude des Pathologies Respiratoires, U1100, F-37032 Tours, France; 2 Université François Rabelais de Tours, F-37032 Tours, France; 3 Plateforme IBiSA de Microscopie Electronique, Université François Rabelais de Tours, F-37032 Tours, France; 4 PRC, INRA, CNRS, Université François Rabelais de Tours, IFCE, F-37380 Nouzilly, France; 5 PAIB, CIRE, INRA, CHRU de Tours, Université François Rabelais de Tours, F-37380 Nouzilly, France; 6 Present Address: Australian Prostate Cancer Research Centre - Queensland, Translational Research Institute, Institute of Health and Biomedical Innovation and School of Biomedical Sciences, Queensland University of Technology (QUT), Brisbane, Australia

## Abstract

Kallikrein-related peptidase 12 (KLK12) is a kallikrein family peptidase involved in angiogenesis – a complex biological process in which the sprouting, migration and stabilization of endothelial cells requires extracellular matrix remodeling. To characterize the molecular mechanisms associated with KLK12′s proangiogenic activity, we evaluated its ability to hydrolyze various matrix proteins. Our results show that KLK12 efficiently cleaved the human extracellular matrix proteins fibronectin and tenascin, both of which are involved in the regulation of endothelial cell adhesion and migration. For fibronectin, the major proteolytic product generated by KLK12 was a 29 kDa fragment containing the amino-terminal domain and the first five type I fibronectin-domains, which are essential for regulating fibronectin assembly. We also demonstrated that KLK12-mediated fibronectin proteolysis antagonizes fibronectin polymerization and fibronectin fibril formation by endothelial cells, leading to an increase in cell migration. Furthermore, a polyclonal antibody raised against KLK12′s proteolytic cleavage site on fibronectin prevented the KLK12-dependent inhibition of fibronectin polymerization and the KLK12-mediated pro-migratory effect on endothelial cells. Taken as a whole, our results indicate that KLK12′s proangiogenic effect is mediated through several molecular mechanisms.

## Introduction

Blood vessel formation (comprising vasculogenesis and angiogenesis) is a critical physiologic process in all human organs, and dysregulation of this process is often observed in human diseases (notably during tumorigenesis)^1–3^. On one hand, vasculogenesis involves the recruitment of progenitor cells (i.e. angioblasts) and their differentiation into endothelial cells (ECs), resulting in the *de novo* formation of blood vessels. On the other hand, angiogenesis corresponds to the extension of pre-existing vessels through EC sprouting, migration and proliferation. These cellular behaviors are tightly controlled in space and time by a complex balance between pro- and anti-angiogenic factors, cell-cell interactions, and cell-extracellular matrix (ECM) interactions^2,3^. Remodeling of the ECM by membrane-bound and extracellular proteases has a central role in blood vessel formation^4^. Firstly, the ECM acts as a reservoir for many pro- and anti-angiogenic factors, and thus proteolysis of ECM components regulates the factors’ bioavailability and activity^5,6^. Secondly, proteolytic processing of ECM proteins affects their integration into the matrix, which in turn directly influences the ECM’s composition and, ultimately, cell-ECM interactions and the associated downstream signaling^7,8^. Lastly, proteolysis of the ECM leads to the generation of bioactive protein fragments (matrikines) and the exposure of cryptic sites in ECM proteins (matricryptic sites/matricryptins), which differ in their biological activity when compared with full-length matrix proteins^9–11^.

Kallikrein-related peptidase 12 (KLK12, a member of the kallikrein-related peptidase family ranging from KLK1 to KLK15) is a secreted serine protease with trypsin-like activity^12,13^. KLK12 is expressed in a large number of human organs and tissues, such as bone, bone marrow, the colon, the lung, the trachea, the prostate, the salivary glands, and the stomach^14^. Expression of KLK12 is dysregulated in several diseases, including breast, gastric and lung cancers^5,6,15,16^. Although KLK12′s biological role is not well understood, several studies have reported a possible involvement in angiogenesis. Most importantly, Giusti *et al*.^17,18^ showed that neutralization of KLK12 altered the formation of tubule-like structures by ECs derived from skin. The mechanism was not fully characterized but may have involved activation of the kinin B2 receptor (B2R). More recently, it was reported that KLK12 also exerts proangiogenic activity towards lung ECs, albeit independently of the B2R signaling pathway^19^. Using biochemical and cell-based assays, it has been shown that KLK12 modulates the bioavailability of platelet-derived growth factor-BB (PDGF-BB) by cleaving a domain that is essential for the factor’s retention in the ECM and/or at the EC surface. Direct and indirect co-culture experiments with ECs and lung stromal cells have demonstrated that the PDGF-BB released from ECs activates the PDGF-receptor-β in stromal cells and thus induces secretion of the proangiogenic factor vascular endothelial growth factor-A (VEGF-A)^6^. Furthermore, it was demonstrated that KLK12 modulates the availability and activity of other angiogenesis-related factors, such as VEGF-A, transforming growth factor-β (TGF-β), bone morphogenetic protein-2 and fibroblast growth factor-2 (FGF-2) through proteolysis of matricellular proteins CCN^5^. The objective of the present study was to identify the molecular mechanisms involved in the regulation of angiogenesis by KLK12. To this end, we investigated KLK12′s ability to hydrolyze various matrix proteins with important roles in angiogenesis, and determined the associated functional consequences.

## Experimental Procedures

### Antibodies and reagents

The monoclonal anti-fibronectin (FN) antibody FN9-1 (directed against the amino-terminal I_1–5_ domains) was obtained from Lonza (Basel, Switzerland). Other monoclonal anti-FN antibodies (IST-2, IST-7, IST-10, and 1C9) were from Sirius Biotech (Genoa, Italia). The polyclonal anti-KLK12 antibody (AF3095), the NorthernLights-493 conjugated donkey polyclonal anti-sheep IgG (NL012), and the phycoerythrin conjugated monoclonal anti-PECAM/CD31 antibody (FAB3567P) were purchased from R&D Systems Europe (Lille, France). Alexa Fluor®-488-conjugated anti-mouse IgG was purchased from Invitrogen (Courtaboeuf, France), and Hoechst compound 33342 was purchased from Interchim (Montluçon, France). The rabbit isotype control was obtained from Cell Signaling (Leiden, Netherlands). Fluorescence resonance energy transfer (FRET) substrate peptides were synthesized by GeneCust (Dudelange, Luxemburg). Purified human matrix proteins were obtained from Abcam (Paris, France) for laminin-5 (Lam-5) and from BD^™^ Bioscience (Le Pont de Claix, France) for plasma FN and collagen IV (coll-IV). Cellular FN (cFN) and EDB-FN (FN-EDB) were purchased from Sirius Biotech (Genoa, Italia). Tenascin-C (Tn-C) was a kind gift from R. Chiquet-Ehrismann, and was produced and purified as previously described (29). NuPage Novex 4–12% Bis-Tris gel, the Coomassie blue staining kit and Silverquest staining reagents were obtained from Invitrogen. Polyclonal antibodies were raised against the KLK12 proteolytic cleavage site on FN (pAb_FN-KLK12_) by immunizing rabbits with KLH-C-SGSGPFTDVRAAVYQ. The antibodies were purified from sera by affinity chromatography (Biotem, France), using the immunizing peptide immobilized on a SulfoLink Resin column (ThermoFisher, France).

### Cells and culture conditions

Human lung blood microvascular ECs (HBMVEC-LBl: tissue acquisition number: 17888; batch number: 123873) were supplied by Lonza. These cells were cultured at 37 °C and 5% CO_2_ in Endothelial Cell Growth Medium 2 containing 5% fetal bovine serum (FBS) and bovine brain extract, human epidermal growth factor, hydrocortisone, gentamicin, amphotericin-B, VEGF, hFGF-B, insulin-like growth factor-1, and ascorbic acid (EGM-MV Bulletkit, Lonza), according to the manufacturer’s instructions. Human lung primary fibroblasts (CCD16-Lu) were obtained from the American Type Culture Collection (LGC Standards Sarl, Molsheim, France) and were cultured in Eagle’s Minimum Essential Medium supplemented with 10% FBS. Unless otherwise specified, all cell treatments were performed in basal medium, and cells were grown for a maximum of seven passages.

### KLK12 activation

Pro-KLK12 was activated according to the manufacturer’s instructions; the enzyme was diluted in activation buffer (100 mM Tris, 150 mM NaCl, 10 mM CaCl2, 0.05% (w/v) Brij35, pH 8.0) and then incubated at 37 °C for 24 h. The KLK12 active site was titrated with plasmin-titrated tissue factor pathway inhibitor 2 (TFPI2), which behaves as a pseudo-irreversible inhibitor of both plasmin and KLK12 (data not shown). KLK12 activity assays and titrations were carried out in activity buffer (TCNB buffer: 100 mM Tris, 150 mM NaCl, 10 mM CaCl2, 0.05% (w/v) Brij35, pH 7.5), using a Boc-VPR-AMC substrate (R&D Systems Europe). Assay buffer alone was used as a negative control for all experiments.

### Digestion of matrix proteins by KLK12

Matrix proteins (TN-C, Lam-5, coll-IV, FN, cFN, and FN-EDB) were diluted in TCNB buffer and co-incubated with active KLK12 at 37 °C. The concentrations of matrix proteins and KLK12, the enzyme:substrate (E:S) ratio and the incubation time are specified in the figure legends. As controls, matrix protein and KLK12 were incubated alone under the same conditions. In control experiments, different protease inhibitors have been used: the cysteine protease inhibitor E-64 (100 µM), the metalloprotease inhibitor Ethylenediaminetetraacetic acid (EDTA, 0.5 mM), the serine protease inhibitor Phenylmethylsulfonyl fluoride (PMSF, 0.5 mM). After incubation, digestion was stopped by adding SDS-PAGE loading buffer, and proteolysis fragments were separated by SDS-PAGE on a NuPage Novex 4–12% Bis-Tris gel under reducing conditions. Migration was performed in MOPS buffer (MOPS 5 mM; Tris 5 mM; EDTA 0.1 mM; SDS 0.01% w/v). Gels were stained with Coomassie Blue or used for Western blots. For Western blots, proteins were transferred onto a polyvinylidene difluoride membrane using Tris/Glycine/SDS liquid transfer (Tris 25 mM; Glycine 192 mM; SDS 0.1% w/v). After transfer and before immunoblotting, membranes were saturated with TBS-T (137 mM sodium chloride, 20 mM Tris, 0.1% Tween-20, buffered at pH 7.6) containing 5% skimmed milk. After immunoblotting, the protein of interest was revealed by chemoluminescence.

### Mass spectrometry

The major proteolytic fragment of FN generated by KLK12 (a 29 kDa band, FN-f) was cut out of the Coomassie-blue-stained gel. Tryptic in-gel digestion (Promega, France) was performed for 16 h at 37 °C, as described elsewhere^20^ Peptide mixtures were analyzed by online nanoflow liquid chromatography - tandem mass spectrometry (nanoLC-MS/MS). The Ettan multidimensional liquid chromatography system (controlled by UNICORN^TM^ software; GE Healthcare, Germany) was used to desalt and separate peptides prior to online MS and MS/MS analyses. Ten µL of digested sample were injected in µL pickup mode. Each sample was automatically desalted and pre-concentrated using a Zorbax 300-SB C18 trap column, 300 µm i.d. x 5 mm (Agilent Technologies, Germany). Peptides were separated on a Zorbax 300-SB C18 column, 75 µm i.d. x 150 mm (Agilent Technologies, Germany). Buffer A consisted of aqueous 0.1% formic acid, and buffer B was 0.1% formic acid in 84% acetonitrile and 16% water. Separation was performed at a flow rate of 350 nL/min, with a 15–55% B gradient for 30 min. Eluted peptides were analyzed online with a LTQ Linear Ion Trap Mass Spectrometer (Thermo Electron, FL, USA) using a Dynamic Nanospray Probe Interface (Thermo Electron). Ionization (1.8–2.1 kV) was performed with a liquid junction and non-coated fused silica nanoESI 25 µm i.d. emitters (New Objective, MA, USA). The ion transfer capillary took place at 200 °C. Each scan cycle consisted of a full-scan mass spectrum (m/z 500–2000) collected in enhanced mode, followed by three MS/MS events in centroid mode (Qz = 0.25, activation time = 40 ms). For collision-induced dissociation spectra (MS2), the isolation width was 2 m/z units, and the normalized collision energy was 40%. Dynamic exclusion was activated to place a mass temporarily onto an exclusion list for 30 s, with a repeat count of 1 (once) in order to analyze less abundant ions with MS/MS. Raw data files were converted to the mzXML format with Bioworks 3.3.1^TM^ software (ThermoFisher Scientific, San Jose, CA). In order to identify the proteins, the peptide and fragment masses obtained were matched automatically against a local database containing the target protein (P02751–7|FINC_HUMAN). MS/MS ion searches were performed using the Mascot Daemon interface and the Mascot search engine (version 2.3, Matrix Science, UK). Enzyme specificity was set to trypsin with two missed cleavages, using carbamidomethylcysteine (+57 Da) and methionine oxidation (+16 Da) as variable modifications. The ion tolerance was set to 1.4 Da for parent ion matches and 1.0 Da for fragment ion matches. Peptides detected with an expected value < 0.05 and a Mascot peptide score >35 were considered to have been positively identified.

### Kinetic measurements

The FRET substrates Abz-GPFTDVRAAVY-(3-NO_2_-Tyr) and Abz-GMQWLKTQGN-(3-NO_2_-Tyr) cover FN residues Gly^284^ to Tyr^294^ (Nter-1) and Gly^319^ to Asn^329^ (Nter-2), respectively. They contain the two potential cleavage sites leading to the release of the 29 kDa FN fragment. Hydrolysis assays were carried out at 37 °C by adding titrated KLK12 (7 nM) to the Nter peptides (0.5 µM) in KLK12 activity buffer. The fluorescence intensity was measured using a spectrofluorimeter (Cary Eclipse, Varian SA, France; excitation wavelength: 320 nm, emission wavelength: 420 nm). The initial rate of hydrolysis (as the fluorescence intensity per minute (Int.min^−1^)) of the Nter-1 peptide by KLK12 was measured with increasing substrate concentrations (from 0 to 200 μM; n = 3). Fluorescence intensity was converted to a molar concentration via a standard curve of serial Abz dilutions. The kinetic parameters K_M_ and k_cat_ were calculated from the experimental curve v = f(S), using the non-linear regression analysis routine in GraphPad Prism software (version 7.00).

### Immunofluorescent staining of fibronectin fibrils formed by endothelial cells

Briefly, 200,000 ECs were seeded in a 6-well plate in complete medium. After a 24-hour incubation, cells were washed and incubated in basal medium. Depending on the experiments, the treatment consisted of FN or FN-EDB (500 nM), 10 nM of active KLK12, and 50 or 500 nM of neutralizing antibody (pAb_FN-KLK12_) or the isotype control antibody. Treatment times are indicated in the figure legends. After treatment, media were harvested, and the cells were washed twice using PBS and fixed in cold methanol for 10 min. Fixed cells were blocked by a 30 min incubation in PBS containing 3% bovine serum albumin (BSA). Fibronectin fibrils were stained with a mouse monoclonal anti-FN antibody (IST-2, 1:200 in PBS + 3% BSA, overnight at 4 °C). After three washes with PBS (5 min at RT), a secondary fluorescent antibody was applied (goat polyclonal anti-mouse IgG antibody - Alexa Fluor®-488, 1:1000 in PBS + 3% BSA, 1 h at RT). Cell nuclei were stained with Hoechst 33342 reagent diluted to 10 µg/ml in PBS for 10 min at room temperature (RT). Stains were visualized using an EVOS FL Cell Imaging System (ThermoFisher, France).

### Differential extraction of fibronectin fibrils formed by endothelial cells

After treatment, FN fibrils were extracted by scraping off the cells and ECM into fresh DOC buffer (1% (w/v) sodium deoxycholate, 20 mM Tris-HCl pH 8.8; 2 mM PMSF; 2 mM EDTA, 2 mM iodoacetic acid, 2 mM N-ethylmaleimide). Next, samples were vortexed and centrifuged at 14,000xg at 4 °C for 30 min. Supernatants were eliminated, and the DOC-insoluble fraction containing FN fibrils was solubilized in SDS buffer (2% (w/v) SDS, 20 mM Tris-HCl pH 8.8; 2 mM PMSF; 2 mM EDTA, 2 mM iodoacetic acid, 2 mM N-ethylmaleimide). The DOC-insoluble fractions and cell supernatants were analyzed by Western blotting, as described above. Western blotting was performed using a mouse monoclonal anti-FN antibody (IST-2) or a mouse monoclonal against FN domains I_1–5_ (FN9–1). The protein of interest was revealed by chemoluminescence.

### The modified Boyden chamber migration assay

Briefly, ECs were suspended in complete medium and placed in the upper chamber on cell culture inserts (pore diameter: 8 μm; Becton Dickinson, France) at a density of 20,000 cells/well. After overnight incubation, the medium in the upper chamber was replaced by basal medium containing 500 nM FN and 10 nM KLK12 or buffer as a control. The bottom chamber was filled with complete medium to stimulate EC migration. After 24 h of incubation at 37 °C and 5% CO_2_, the cells that had migrated to the lower side of the filter and the bottom chamber were fixed with cold methanol (10 min at RT) and stained with Hoechst 33342 diluted to 10 µg/ml in PBS during 10 min at room temperature. Migrated cells were visualized using an EVOS FL Cell Imaging System (ThermoFisher, France) and automatically counted using ImageJ software (https://imagej.nih.gov/ij/).

### Time-lapse video microscopy

Briefly, 100,000 ECs per well were plated in a 35 mm glass-bottomed dish (MatTek, Ashland, MA, USA) in complete medium. Twenty-four hours after plating, cell nuclei were stained by 30 min of treatment with Hoechst 33342 reagent diluted to 1 µg/ml in complete culture medium. Before treatment, cells were washed with basal medium and incubated for 1 h in a microscope incubator (37 °C, 5% CO_2_). The cells were treated with 10 nM KLK12, 500 nM FN, 500 nM neutralizing antibody (pAb_KLK12-FN_) or the isotype control or the same volume of KLK12 activity buffer (the control condition). Migration of ECs was monitored at 37 °C and 5% CO_2_ for 5 h, using confocal microscopy (Olympus IX81-ZDC, 20X lens) which imaged cells at 5 min interval_._ Cells were tracked using Imaris Software (Bitplane, Zurich, Switzerland), based on Hoechst staining. Only cells tracked during the 5 h period were included in the analysis. For each cell, displacement length and velocity were calculated over the 5 h period. At least 40 cells were analyzed for each treatment condition.

### Immunofluorescent staining of KLK12 and fibronectin in the capillary formation assay

Capillary formation assay was performed as previously described^6^. Briefly, ECs and fibroblasts were seeded together (20,000 cells/well of each cell type) in a 24-well plate in complete EC medium. After 9 days of culture (medium renewal: every 48 h), the cells were washed twice using PBS and fixed in cold methanol for 10 min. Fixed cells were blocked by 30 min of incubation in PBS containing 3% BSA. Fibronectin was stained with a mouse monoclonal anti-FN antibody (IST-2, 1:200 in PBS + 3% BSA, overnight at 4 °C). KLK12 was stained with a sheep polyclonal anti-KLK12 antibody (AF3095, 1:100 in PBS + 3% BSA, overnight at 4 °C). After three washes with PBS (5 min at RT), a secondary fluorescent antibody was applied (goat polyclonal anti-mouse IgG antibody - Alexa Fluor®-488 or donkey polyclonal anti-sheep IgG – NorthernLights-493, 1:1000 in PBS + 3% BSA, 1 h at RT). PECAM/CD31 was stained using an anti-CD31 antibody conjugated to phycoerythrin (FAB3567P, 1:100 in PBS + 3% BSA, overnight at 4 °C). Cell nuclei were stained with Hoechst 33342 reagent (diluted to 10 µg/ml in PBS) for 10 min at RT. Staining was visualized using a confocal microscope (Olympus IX81-ZDC, 20x and 40x lens). The resulting Z-stack images were superposed using Imaris Software, and maximum intensity projection images are presented (Bitplane, Zurich, Switzerland).

### Statistical analysis

Non-parametric statistical analyses were performed with using GraphPad Prism software (version 7.00). All results are expressed as the mean ± standard deviation (SD) and, unless otherwise specified, represent at least three independent experiments.

## Results

### Proteolysis of ECM proteins by KLK12

**Figure 1:**
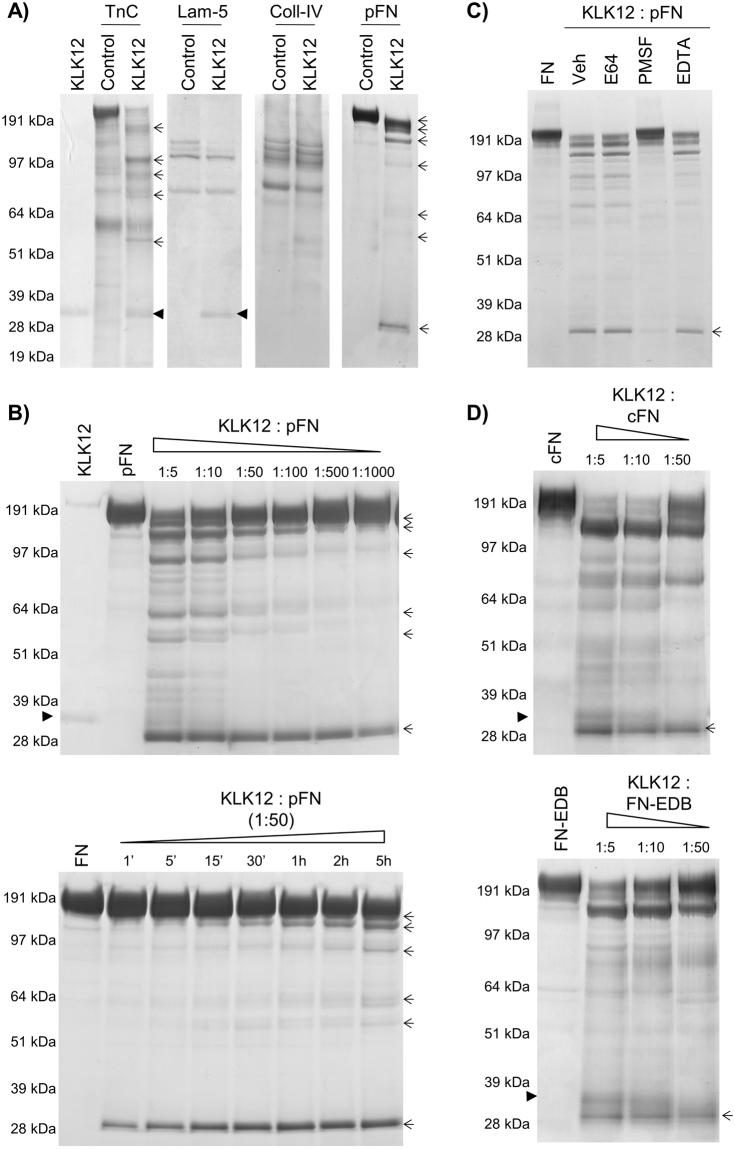
KLK12 exerts proteolytic activity against fibronectin isoforms. (**A**) Proteolysis of matrix proteins by KLK12, analyzed with SDS-PAGE and Coomassie blue staining (E:S molar ratio: 1:5, 4 h at 37 °C). (**B**) Characterization of FN proteolysis by KLK12, analyzed as in A. *Left panel:* Dose-dependent proteolysis of FN by KLK12 with an E:S molar ratio ranging from 1:5 to 1:1000; 4 h at 37 °C. *Right panel:* Time-dependent proteolysis of FN by KLK12, from 1 min to 5 h at 37 °C (E:S molar ratio: 1:50). (**C**) In control experiments, KLK12 was incubated with either E64 (100 µM), PMSF (0.5 mM) or EDTA (0.5 mM) and with FN. (**D**) Dose-dependent proteolysis of cellular FN isoforms (cFN and FN-EDB) by KLK12. E:S molar ratio: from 1:5 to 1:50; 4 h at 37 °C. The band corresponding to recombinant human KLK12 is indicated by an arrowhead when visible, and bands corresponding to proteolytic products are indicated by arrows.

To determine the proteolytic activity of KLK12 towards ECM proteins, we tested KLK12′s ability to cleave Tn-C, Lam-5, coll-IV and plasma FN in biochemical assays. Our results showed that under our experimental conditions (E:S = 1:5, 4 h at 37 °C), KLK12 was able to efficiently cleave Tn-C and FN only (Fig. 1A). After incubation of Tn-C or FN with KLK12, several proteolytic products were visualized (indicated by arrows in Fig. 1A), with apparent masses ranging from 190 kDa to 30 kDa (for Tn-C) and to 29 kDa (for FN). An analysis of dose-dependent proteolysis of FN by KLK12 (E:S from 1:5 to 1:1000; 4 h at 37 °C) demonstrated that KLK12 is able to cleave FN even at very low E:S ratio - generating a main proteolytic product with an apparent mass of 29 kDa (Fig. 1B). Addition of the synthetic serine protease inhibitor PMSF reduced proteolysis of FN by KLK12, while inhibitors of other protease classes had no effect (Fig. 1C). A time-dependent experiment showed that KLK12 rapidly (after 1 min of co-incubation) released a proteolytic product with an apparent molecular mass of 29 kDa (E:S ratio: 1:50, Fig. 1B and D), which accumulated over time. Lastly, we evaluated KLK12′s proteolytic activity for cFN (a mixture of FN variant proteins expressed in different tissues) and FN-EDB (associated with vessel formation). Interestingly, KLK12 was also able to generate the 29 kDa FN proteolytic fragment (FN-f) from cFN variants, including FN-EDB.

### Identification of the major proteolytic product formed by KLK12 from fibronectins

**Figure 2:**
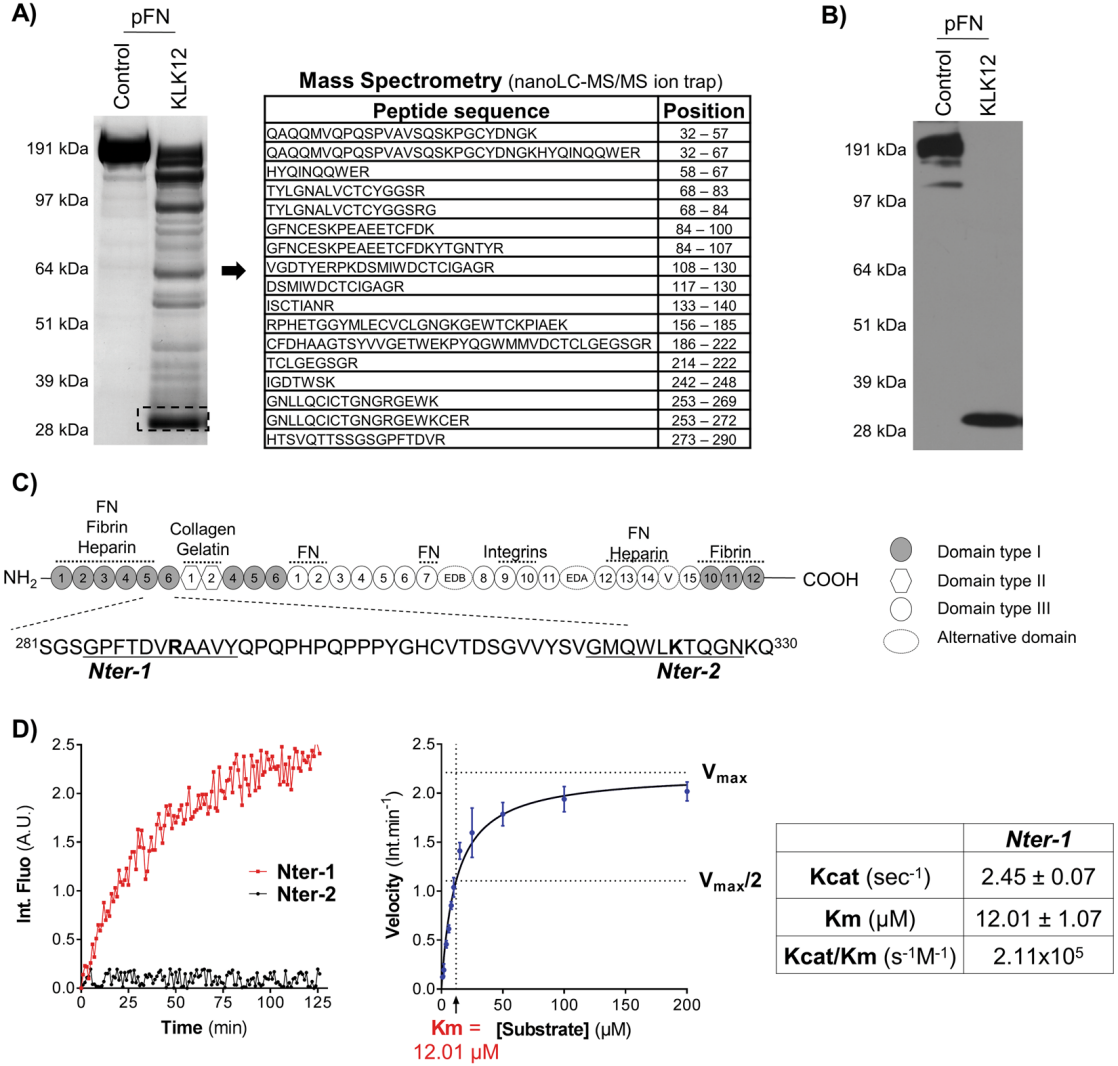
KLK12 releases the amino-terminal domains of fibronectin. (**A**) Characterization of the 29 kDa proteolytic fragment of FN (FN-f) generated by KLK12. After proteolysis of pFN by KLK12 (E:S molar ratio: 1:50; 4 h at 37 °C), FN proteolytic fragments were separated by SDS-PAGE, and the 29 kDa fragment was analyzed using mass spectrometry. The sequences and position of peptides corresponding to the amino-terminal domains of FN are indicated. MS/MS results are summarized in Supp Table 1. (**B**) Western blot of FN proteolytic fragments generated by KLK12 (as in A), using an FN9-1 antibody directed against the amino-terminal domains of FN. Western blots with other anti-FN antibodies are shown in Supp. Figure 1. (**C**) A schematic depiction of the domain structure of FN. Intra- and intermolecular interactions are indicated above the diagram. The sequences of the two potential KLK12-cleavage sites (leading to the release of the 29-kDa fragment) are indicated below the diagram (*Nter-1* and *Nter-2*). (**D**) Left panel: hydrolysis of Nter-1 and Nter-2 FRET peptides (0.5 µM) by KLK12 (7 nM). Right panel: non-linear regression curve for hydrolysis of the Nter-1 peptide by KLK12. The initial velocity (in fluorescence intensity per minute (Int.min^−1^)) of the E:S reaction was measured with increasing S concentrations (0–200 μM). Table: Kinetic constants calculated for the Nter-1 peptide in a non-linear regression analysis.

To identify KLK12′s main cleavage site on FN, we characterized the 29 kDa FN-f. Firstly, tandem mass spectrometry analysis resulted to the identification of 17 peptides corresponding to the amino-terminal region of FN (32–290) (supplementary Table 1), which is common to all the different FN variants (Fig. 2A) and contains the five first type I FN domains (I_1–5_). Secondly, we performed Western blots using a set of four antibodies recognizing different domains of FN (Supplementary Figure 1); these experiments showed that the 29 kDa FN-f is only detected by an antibody directed against FN’s amino-terminal domains (Fig. 2B). Based on the tandem mass spectrometry analysis, KLK12 might cleave FN at two potential sites: Arg^290^ or Lys^324^. To identify the main KLK12-cleavage site, we designed FRET peptides covering the sequences of the two potential cleavages sites (Fig. 2C): from Gly^284^ to Tyr^294^ (*Nter-1* peptide) and Gly^319^ to Asn^329^ (*Nter-2* peptide). Hydrolysis studies showed that only the *Nter-1* peptide was efficiently hydrolyzed by KLK12. In a nonlinear regression analysis, we determined the Michaelis constant (K_m_ = 12.01 µM) and the turnover number (k_cat_ = 2.45 sec^−1^) for KLK12′s hydrolysis of the *Nter-1* peptide; this allowed us to calculated the second-order rate constant (k_cat_/K_m_ = 2.11 × 10^5^ s^−1^.M^−1^, Fig. 2D). Taken as a whole, these results show that the 29 kDa FN-f contains the I_1–5_ domains of FN and terminates at Arg^290^.

### Effect of KLK12 on the polymerization of fibronectin and on fibronectin fibril formation by endothelial cells

During ECM assembly, soluble FN is converted into a dense, insoluble fibril network by polymerization of FN via a cell-dependent molecular process^21,22^. Given that the I_1–5_ FN domains are essential for FN-fibril formation^23^, we speculated that KLK12-mediated FN proteolysis and generation of 29 kDa FN-f would limit FN-fibril formation.

**Figure 3:**
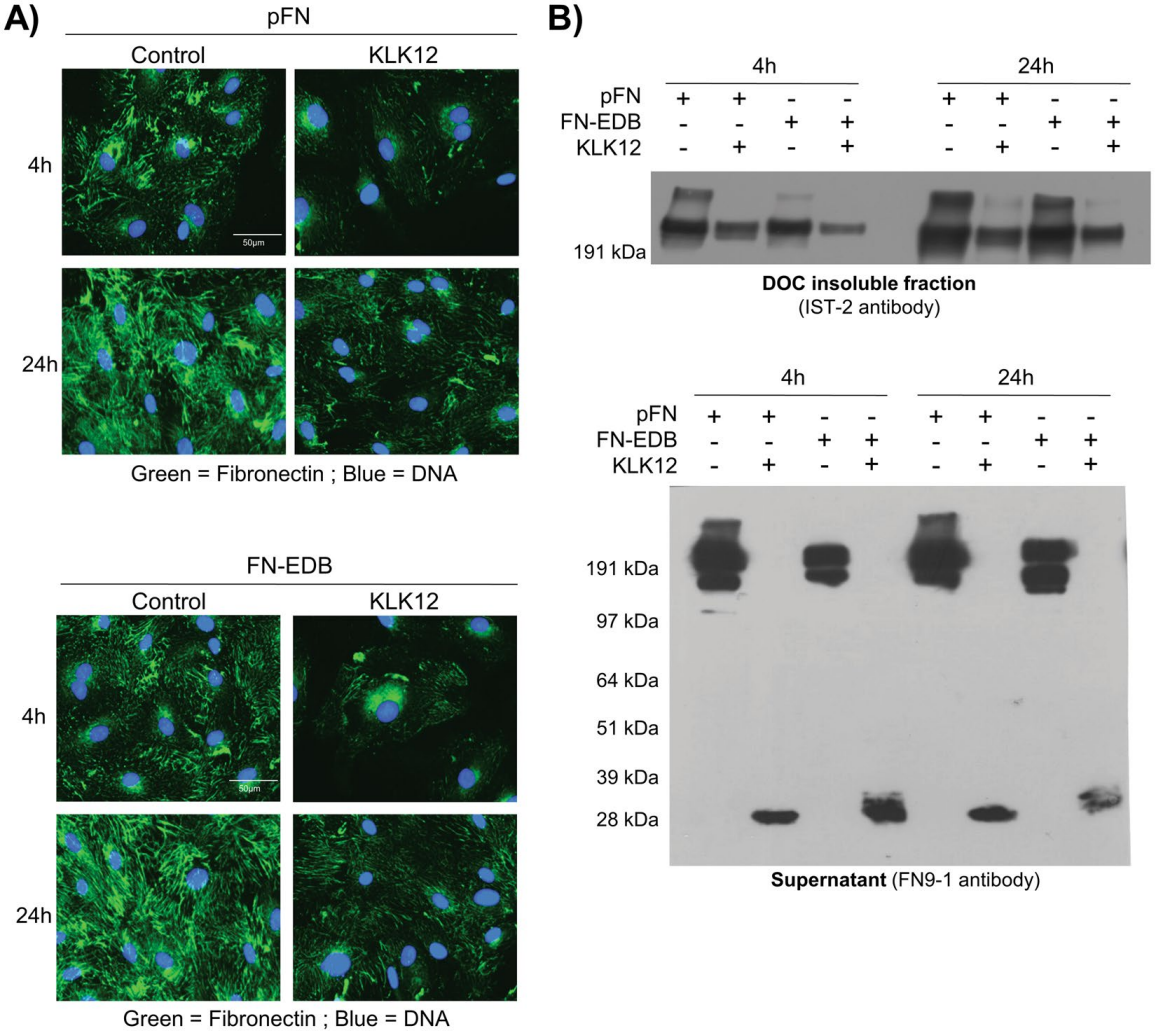
KLK12 modulates fibronectin matrix assembly by endothelial cells. The impact of KLK12 on FN matrix assembly by human ECs was analyzed after treatment with 10 nM KLK12 for 4 h or 24 h in basal medium containing 500 nM FN or FN-EDB. (**A**) Immunofluorescence analysis. After treatment, cells and matrix were fixed, FN fibrils were immunodetected (monoclonal anti-FN antibody IST-2, green channel), and DNA was stained using Hoechst reagent (blue channel). Scale bar = 50 µm. (**B**) Western blot. After treatment, the DOC-insoluble protein fraction and conditioned media were harvested and analyzed by Western blotting. Upper panel: Detection of FN fibrils in the DOC-insoluble protein fraction using the IST-2 anti-FN monoclonal antibody. Lower panel: Detection of soluble FN in conditioned media using the FN9-1 antibody directed against the amino-terminal domains of FN. The sizes of the protein markers (in kDa) are indicated on the left.

To evaluate KLK12′s impact on FN-fibril formation, we first performed immunofluorescence staining of FN-fibrils formed by ECs after treatment with KLK12 in the presence of exogenous FN. Our results show that (i) FN (either 500 nM FN or FN-EDB) was rapidly incorporated into the ECM, where it formed fibrils and (ii) KLK12 (10 nM) significantly decreased the formation of FN-fibrils (Fig. 3A). Secondly, we performed a Western blot analysis after the differential solubilization of soluble and polymerized FN using deoxycholate (DOC) buffer (enabling the fractionation of DOC-soluble and DOC-insoluble FN-fibrils). The Western blots showed that a lower amount of FN-fibrils was incorporated into the ECM in the presence of KLK12 (10 nM) (Fig. 3B, upper panel). Moreover, Western blots of the supernatant showed that the 29 kDa FN-f was released by KLK12 in these cell-based experiments (Fig. 3B, lower panel).

### KLK12′s impact on fibronectin fibril formation can be abrogated by a polyclonal antibody against the KLK12-cleavage site on fibronectin

**Figure 4:**
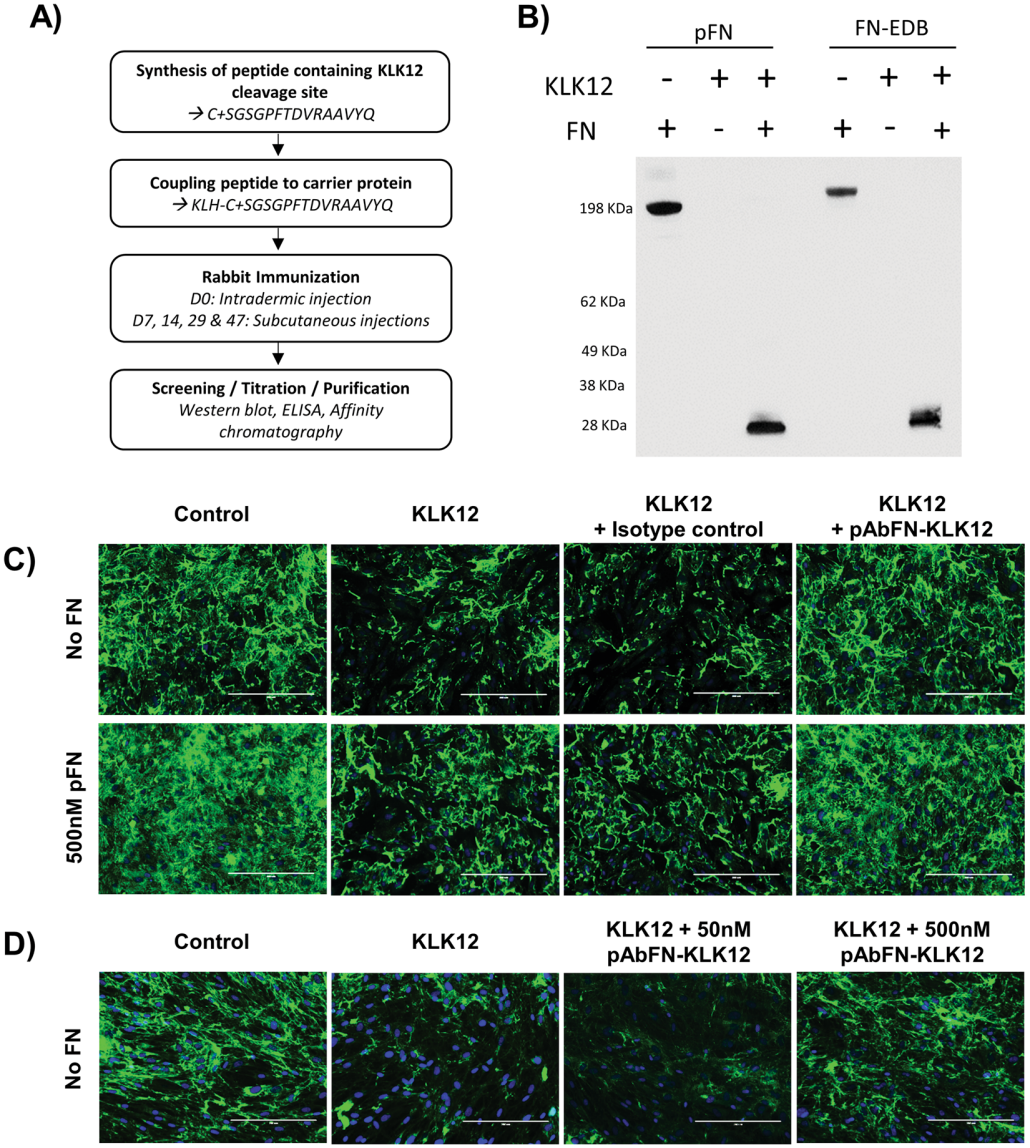
KLK12 neutralization modifies fibronectin matrix assembly by endothelial cells and fibroblasts. (**A**) Description of the protocol used to generate pAbFN-KLK12, an antibody that recognizes the KLK12 cleavage site. (**B**) Test of pAbFN-KLK12′s specificity on KLK12-generated FN fragments. KLK12-generated proteolytic fragments of FN and FN-EDB were prepared as in Fig. 2A and then analyzed in Western blot. (**C** and **D**) The impact of KLK12 neutralization on FN matrix assembly by ECs (**C**) and fibroblasts (**D**), using a neutralizing antibody that targeted the KLK12 cleavage site on FN (pAbFN-KLK12). Human ECs were treated with 10 nM KLK12 for 24 h in basal medium with or without FN (500 nM) and pAbFN-KLK12 or the isotype control (500 nM). Fibroblasts were treated with 10 nM KLK12 for 24 h in basal medium with or without pAbFN-KLK12 (50 nM and 500 nM). After treatment, cells and matrix were fixed, the FN fibrils were immunodetected (monoclonal anti-FN antibody IST-2, green channel) and DNA was stained using Hoechst reagent (blue channel). Scale bar = 200 µm.

We developed a polyclonal antibody against KLK12-cleavage site on FN (pAb_FN-KLK12_, Fig. 4A) that recognizes full-length FN, the FN-EDB variant, and the 29 kDa FN-f (Fig. 4B). To test the antibody’s ability to limit KLK12-mediated FN proteolysis, we analyzed FN-fibrils formed by ECs treated with KLK12 (10 nM) in the presence or absence of exogenous FN, a control antibody (isotype control) or pAb_FN-KLK12_. Immunofluorescent staining of FN showed that, as previously observed, ECs rapidly incorporated soluble FN into their ECM (where FN-fibrils are formed), and that KLK12 decreased FN-fibril formation (regardless of whether they were generated from endogenous or exogenous FN). However, in the presence of pAb_FN-KLK12_, the impact of KLK12 on FN-fibril formation was greatly diminished, and the isotype control antibody did not modulate KLK12′s effect on FN-fibril formation (Fig. 4C). The neutralizing antibody’s effect on the KLK12-mediated decrease in FN-fibril formation was also confirmed in experiments with fibroblasts, which secrete large quantities of FN (Fig. 4D).

### KLK12 stimulates the migration of endothelial cells and suppresses the adhesive properties of fibronectin

**Figure 5:**
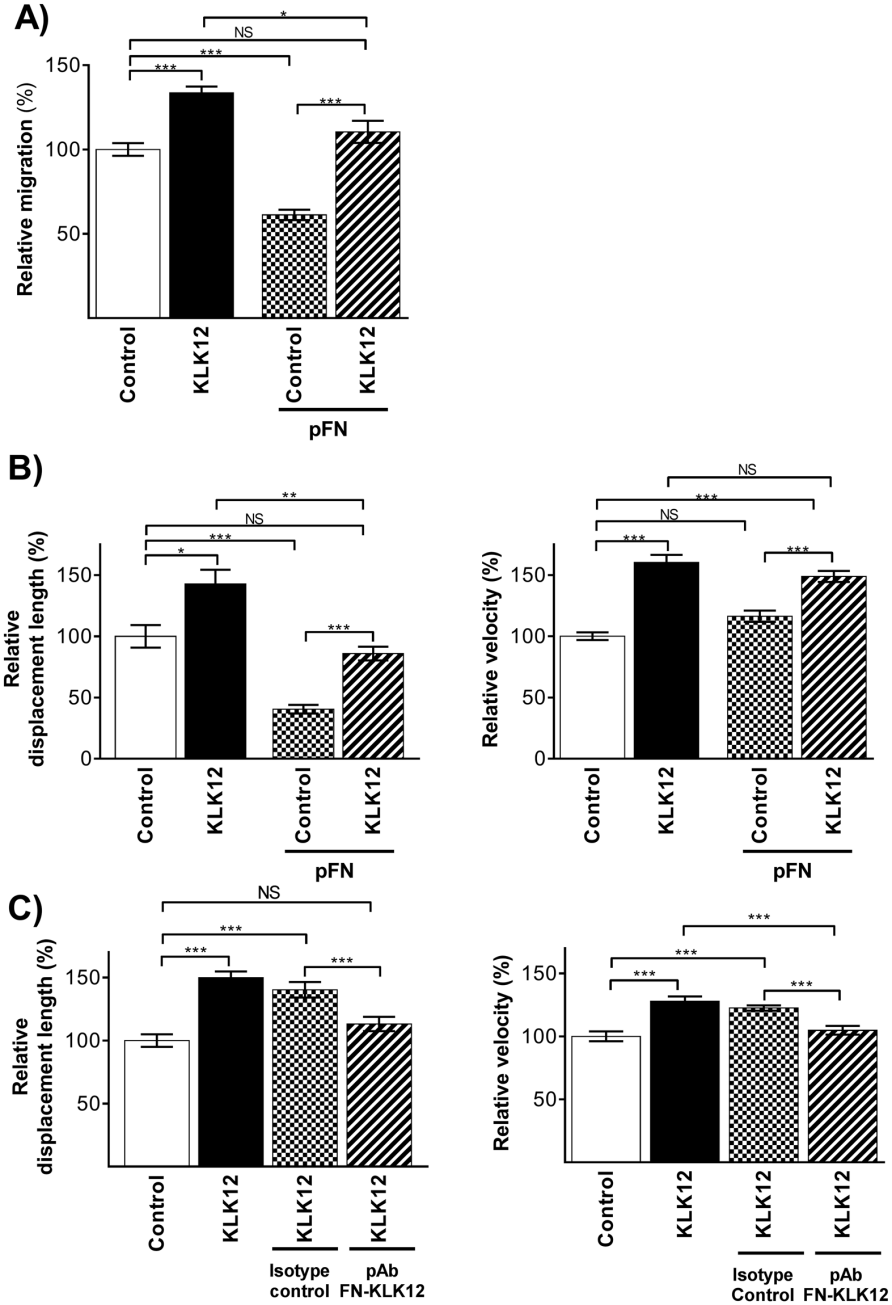
Impact of KLK12 on the migration of lung-derived endothelial cells: (**A**) Endothelial cell migration was analyzed in a Boyden chamber assay after treatment with 500 nM FN in the presence or absence of 10 nM KLK12. After 24 h, migrated cells were stained with DAPI and counted. (**B**) Endothelial cell migration was analyzed by time lapse video microscopy after treatment with 10 nM KLK12 in the presence or absence of 500 nM FN (5 h at 37 °C, 5% CO_2_). At least 40 cells per condition were analyzed on a total of three biological replicates. Cells were tracked (using Imaris software) on the basis of nuclear staining and bright field images, and the displacement length (left) and velocity (right) were determined. (**C**) Endothelial cell migration after treatment with 10 nM KLK12 in the presence or absence of 500 nM of isotype control antibody or pAbFN-KLK12 targeting the KLK12-cleavage site on FN, according to the protocol described in B. *p < 0.05, **p < 0.01, ***p < 0.001 in a two-way Kruskal-Wallis analysis of variance.

Fibronectin fibrils are involved in regulation of the balance between cell adhesion and cell migration^22–26^. To determine whether or not disruption of FN-fibrils by KLK12 could affect this balance, we studied the enzyme’s impact on EC migration in the presence and absence of exogenous FN. Firstly, we used a modified Boyden chamber assay to show that KLK12 treatment (10 nM) of ECs significantly increased their migration (+1.33-fold vs. the control), whereas treatment with exogenous FN (500 nM) significantly decreased EC migration (−1.39-fold vs. the control) (Fig. 5A). As expected, KLK12 treatment suppressed the pro-adhesive effect of exogenous FN, and restored EC migration (Fig. 5A). Secondly, we performed real time migration assays using time-lapse video microscopy on ECs treated with KLK12 in the presence or absence of exogenous FN. As shown in Fig. 5B, KLK12 exerted a pro-migratory effect on EC and increased their displacement length (+1.42-fold vs. the control), whereas exogenous FN reduced EC displacement (-1.60-fold vs. the control; Fig. 5B, Left). KLK12 suppressed the effect of exogenous FN on EC displacement. Interestingly, only KLK12 treatment significantly increased the ECs’ mean velocity (+1.60-fold and +1.48-fold in absence and presence of exogenous FN, respectively, vs. the control condition: Fig. 5B, Right). Lastly, with a view to establishing a link between KLK12-mediated FN proteolysis and the pro-migratory effect of KLK12, similar experiments were performed in the presence of the neutralizing pAb_FN-KLK12_ or an isotype control. As previously observed, KLK12 increased both the displacement and velocity of the ECs (+1.48- and +1.27-fold, respectively, vs. the control; Fig. 5C). Interestingly, KLK12′s effect was totally inhibited in the presence of pAb_FN-KLK12_, (the antibody that also neutralized KLK12′s effect on FN-fibril formation).

### In a capillary formation assay, fibronectin and KLK12 are localized around capillaries formed by endothelial cells

**Figure 6:**
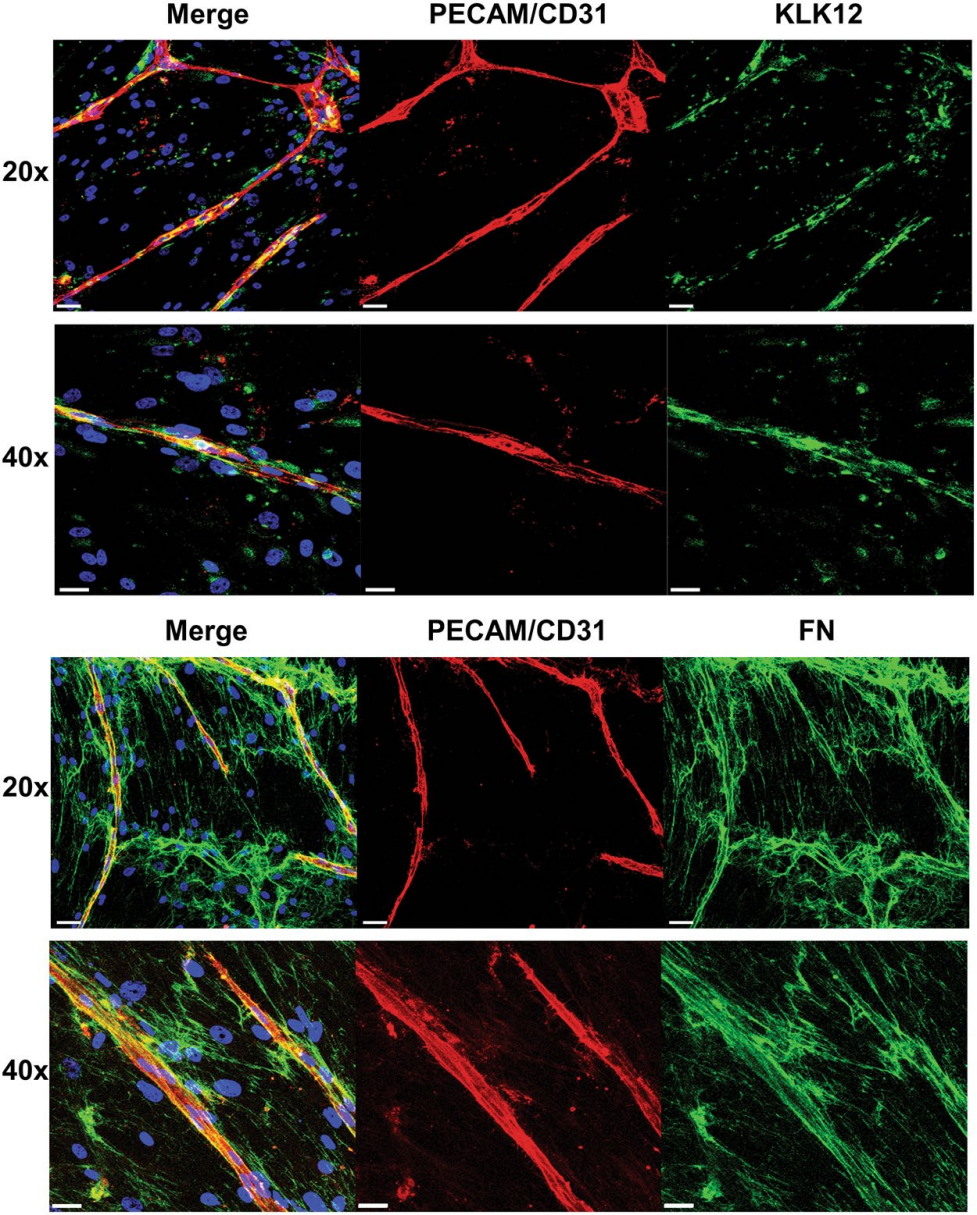
Fibronectin and KLK12 are both expressed in the vascular endothelium. Immunofluorescent staining of capillaries formed after 9 days of fibroblast-EC co-culture. Top panel: Staining of KLK12 (green), PECAM/CD31 (red), and nuclei (blue). Bottom panel: Staining of FN (green), PECAM/CD31 (red), and nuclei (blue). Colocalization of KLK12 or FN with capillaries (CD31/PECAM staining) appears in yellow. Scale bars correspond to 50 µm for 20x images and 30 µm for 40x images.

To determine whether or not KLK12-mediated FN proteolysis could occur during angiogenesis, we investigated the localization of FN and KLK12 in a three-dimensional capillary formation assay described in detail elsewhere^6^. After 9 days of co-culture with fibroblasts, staining of the ECs with PECAM/CD31 showed that the cells had formed capillaries, thereby mimicking angiogenesis. High levels of FN were produced in this model, forming very dense FN fibrils (Fig. 6, bottom panel). Interestingly, the dense FN fibrils were co-localized with PECAM/CD31 staining (Fig. 6, bottom panel), which suggests that this ECM protein is involved in angiogenesis. KLK12 was detected around both ECs and fibroblasts in this co-culture assay. More importantly, and as observed for FN, intense KLK12 staining was seen around the capillaries formed by ECs - suggesting that KLK12 is involved in remodeling of the ECM near to capillaries (Fig. 6, Top panel).

## Discussion

Angiogenesis is a physiologic cellular process that occurs in almost all human tissues and at all stages of life (from the embryonic stage to old age). The process involves complex cellular events leading to the formation of new blood vessels from existing ones. The ECM is known to contribute to angiogenesis in many ways^1,4,27^. Furthermore, angiogenesis is also often dysregulated in pathological conditions and notably during cancer, since tumors need to trigger the formation of new blood vessels in order to gain access to the essential nutrients and oxygen required for growth^2^. Several members of the kallikrein-related peptidase family are reportedly involved in angiogenesis^28,29^. For example, KLK3 (also known as prostate-specific antigen) exerts an anti-angiogenic activity, supposedly by generating anti-angiogenic peptides^30,31^. Conversely, proangiogenic activity has been reported for KLK12, which is involved in the regulation of various molecular mechanisms controlling angiogenesis. KLK12 can directly or indirectly activate EC receptors, such as the kinin receptor B2R. Furthermore, it regulates the bioavailability and activation of growth factors such as PDGF-BB, VEGF-A and FGF-2 through either direct cleavage of a growth-factor-retention domain or degradation of the ECM^5,6,18,19^. Given that (i) kallikreins are well known for their proteolytic activity toward ECM proteins, and (ii) ECM remodeling has an essential role during angiogenesis, we hypothesized that the proangiogenic properties of secreted KLK12 are also related to its proteolysis of ECM proteins.

Hence, we assessed the KLK12′s proteolytic activity toward several ECM proteins involved in angiogenesis (Tn-C, Lam-5, Coll-IV and FN) using *in vitro* biochemical assays with recombinant human KLK12 and purified ECM proteins. We found that KLK12 efficiently cleaves Tn-C and FN but not Lam-5 or Coll-IV. Interestingly, Tn-C and FN share a number of structural and biological characteristics^32^. They are both large modular ECM glycoproteins formed by disulfide-linked subunits. Furthermore, Tn-C and FN contain several structural domains, including a succession of FN type III domains that accounts for at least half of each molecule. After binding to cell receptors, both Tn-C and FN assemble into an insoluble matrix that influences the assembly of other matrix proteins and the latter’s integration into the ECM. Lastly, the expression of these proteins in the vasculature is often dysregulated following vessel injury and pathologic angiogenesis^27^. In the present study, we chose to focus on the function of KLK12-mediated FN proteolysis in angiogenesis; however, considering that both Tn-C and FN are key players in blood vessel formation, it would certainly be useful to evaluate the impact of KLK12-mediated proteolysis of Tn-C on this process.

Two forms of FN are present in human body, a soluble form found at high levels in the blood (plasma FN, mainly produced by hepatocytes) and cFN (produced by several cell types in tissues, where it is incorporated into the ECM)^23,32,33^. The major difference between plasma FN and cFN is the presence of three alternative domains in cFN as a result of alternative splicing of FN mRNA: the highly conserved FN type III extra domains A and B (EDA and EDB) and the non-homologous variable domain. Fibronectin variants containing extra domains are known to be expressed in specific tissues, where they exert specific functions. For example, FN containing EDA and EDB domains (also referred to as oncofetal FN variants) are associated with angiogenesis, and are often quantified as a marker of neovessel formation in the tumor microenvironment^33–36^. In the present study, we showed that KLK12 efficiently cleaves both FN and cFN (and particularly FN-EDB), regardless of whether they were in soluble form or incorporated into the ECM. Proteolysis of FN by KLK12 generates many proteolytic products, although some are only produced from a specific FN isoform. This suggests that some KLK12-cleavage sites might be cryptic in cFN variants, as has already been demonstrated for antibodies recognizing epitopes located within cryptic sites in FN-EDB^37^. However, the major product of FN proteolysis by KLK12 (the 29 kDa FN-f) was produced from all isoforms. As demonstrated by a variety of analytical approaches, this proteolytic fragment corresponds to the amino-terminal part of FN containing the domains I_1–5_, and ends at Arg^290^. This part of FN is common to all FN variants, and corresponds to the heparin-binding domain; the latter is also involved in the FN-TN-C interaction^32,33^. FN-f is soluble, and is detected in the supernatant of ECs after KLK12 treatment. Interestingly, a similar cleavage site on FN has been identified for other trypsin-like proteases, such as the metalloprotease-disintegrin ADAM8, thrombin and KLK14^38–40^.

As mentioned above, FN has a key role in regulation of the ECM architecture through its many interactions with other matrix components, soluble factors, and cellular receptors^7,26,33^. These interactions are modulated by conformational changes in FN, which are controlled by the interactions between FN dimers and the interactions between FN dimers and cell receptors, such as integrins^41,42^. In the ECM, the polymerization of several FN dimers generates insoluble fibrils via a complex, cell-dependent mechanism. This conformational change involves the Arg-Gly-Asp (RGD) cell-binding sequence located in the FN domain III_10_, together with the nearby synergy site located in the FN domain III_9_ that enhances the binding of integrin receptors to RGD. Next, the mechanical forces applied to FN dimers by the integrin-actin cytoskeleton induce a conformational change in FN, which modifies the accessibility of several FN domains required for FN polymerization and fibril assembly^40,41^. The amino-terminal domains I_1–5_ are among the most important domains for FN assembly. They interact with other FN domains (such as domains III_1_ and III_12–14_) and thus allow the polymerization of FN into fibrils^22,23,42^. Here, we found that KLK12 cleaves FN’s I_1–5_ domains, and thus impairs the assembly of soluble FN into insoluble FN fibrils. By adding a polyclonal antibody directed against the region comprising the KLK12-cleavage site (pAb_FN-KLK12_) located after Arg^290^ on FN (leading to the release of the 29 kDa fragment), we showed that blocking this site limits KLK12′s effect on FN fibril formation in cell-based experiments. Given the I_1–5_ domains’ essential role in FN assembly, it is also possible that the soluble 29 kDa FN fragment released by KLK12 might compete with the I_1–5_ domains of intact FN dimers and thus inhibit FN fibril formation, as has been previously suggested^42,43^. Therefore, KLK12 alters FN assembly and causes FN-matrix disruption both directly (by degrading FN) and indirectly (by releasing the I_1–5_ domains, which could interfere with FN polymerization). Although KLK12 efficiently cleaved FN in biochemical assays, we cannot rule out an indirect effect of KLK12 and the involvement of other proteases in KLK12-mediated FN-fibril proteolysis. Indeed, KLK12 possesses trypsin-like activity that might activate many other FN-degrading pro-enzymes. For example, several FN-degrading members of the kallikrein-related peptidase family (such as KLK1-3, KLK5, KLK13 and KLK14^12^) are reportedly activated by KLK12^44^.

Many of FN’s biological functions depend on the protein’s assembly into a functional fibrillar matrix^26,27,33^. In particular, FN fibrils have been implicated in angiogenesis (and especially the regulation of EC adhesion and migration), although the reported effects are subject to debate^24,45,46^. In the present study, we showed that KLK12 treatment of ECs was associated with increases in the mean displacement and mean velocity. Moreover, exogenous FN reduced the displacement of ECs, and this effect could partially be inhibited by KLK12 co-treatment. Lastly, by using the neutralizing polyclonal antibody pAb_FN-KLK12_, we showed that inhibition of KLK12-mediated FN proteolysis significantly reduces the protease’s pro-migratory effect. These various observations support the hypothesis whereby (i) FN reduces EC migration by forming a dense ECM and thus increasing cell adhesion and, conversely, (ii) KLK12-induced proteolysis of FN reduces the formation of a dense ECM and thus promotes EC migration. This hypothesis is also supported by a recent study from Chung *et al*., who found an inverse correlation between the migration of ECs seeded on porous substrates and the length of FN fibrils^46^. Interestingly, a similar mechanism has been reported for other proteases, such as the membrane type metalloproteinase 1 (MT1-MMP/MMP14). For example, MT1-MMP promotes the extracellular proteolysis of FN, regulates FN polymerization and FN fibril turnover, and accelerates the migration of myofibroblasts^47^. Accordingly, KLK12 may also regulate the migration of other cell types via ECM remodeling. In a recent study with Transwell experiments, Zhao *et al*. demonstrated that KLK12 enhanced the migration of gastric cancer cells^48^. However, KLK12 was not able to modulate gastric cancer cell migration in the presence of basement membrane extracts containing mainly laminin and collagen IV, which are not cleaved by KLK12.

The proangiogenic effect of KLK12 (mediated by both its impact on the ECM and on the bioactivity of proangiogenic factors^5,6^) is relevant because of its over-expression in several diseases in which angiogenesis is dysregulated. For example, KLK12 is overexpressed in lung and gastric tumors^5,6,15^, and it is now well established that the formation of new blood vessels in the vicinity of a tumor is critical for the latter’s development. Inversely, KLK12 expression is downregulated in ECs from patients with systemic sclerosis, which is associated with abnormal angiogenesis^17,18^. In the present study, we used a capillary formation assay^6^ to show that FN fibrils are located around capillaries formed by ECs - as observed *in vivo*. Interestingly, KLK12 staining was also observed around capillaries, which suggests that KLK12 might be in contact with FN during capillary formation and thus would be able to exert its proteolytic activity. This finding further supports a potential role for KLK12 in angiogenesis.

In conclusion, our present findings complement previous reports on the multifaceted role of KLK12 in angiogenesis. We demonstrated here that the ECM remodeling initiated by KLK12 impairs and disrupts the FN matrix and promotes EC migration, which is a critical event in the formation of new blood vessels. Moreover, neutralizing KLK12-mediated FN proteolysis with a polyclonal antibody directed against the KLK12 cleavage site (pAb_FN-KLK12_) inhibited the protease’s effect on EC migration. It would therefore be interesting to evaluate the therapeutic potential of this antibody in diseases in which both KLK12 and angiogenesis are dysregulated.

## Electronic supplementary material

Supplementary information 1

Supplementary dataset 1
