# Supplementary material for: Human kallikrein-related peptidase 12 stimulates endothelial cell migration by remodeling the fibronectin matrix
Source: Sci Rep. 2018 Apr 20;8:6331. doi: 10.1038/s41598-018-24576-9 (PMC5910384; doi:10.1038/s41598-018-24576-9)
Supplement: Supplementary file 1 — Supplementary information 1 [file 41598_2018_24576_MOESM1_ESM.docx]

**Human kallikrein-related peptidase 12 stimulates endothelial cell migration through remodeling the fibronectin matrix.**

# Kryza T.^1,2*^, Parent C.^1,2**^, Pardessus J.^1,2**^, Petit A.^1,2^[, Burlaud-Gaillard J.^2,3^](https://www.ncbi.nlm.nih.gov/pubmed/?term=Chiquet-Ehrismann%20R%5BAuthor%5D&cauthor=true&cauthor_uid=25999144), Reverdiau P.^1,2^, Iochmann S.^1,2^, Labas V.^4,5^, Courty Y.^1,2^, Heuzé-Vourc’h N.^1,2^

^1^ INSERM, Centre d'Etude des Pathologies Respiratoires, U1100, F-37032 Tours, France

^2^ Université François Rabelais de Tours, F-37032 Tours, France

^3^ Plateforme IBiSA de Microscopie Electronique, Université François Rabelais de Tours, F-37032 Tours, France

^4^ PRC, INRA, CNRS, Université François Rabelais de Tours, IFCE, 37380 Nouzilly, France.

^5^ PAIB, CIRE, INRA, CHRU de Tours, Université François Rabelais de Tours, 37380 Nouzilly, France.

* Current affiliation: Australian Prostate Cancer Research Centre - Queensland, Translational Research Institute, Institute of Health and Biomedical Innovation and School of Biomedical Sciences, Queensland University of Technology (QUT), Brisbane, Australia.

** CP and JP equally contributed to this work

**Key words:** extracellular matrix processing, fibronectin assembly, fibronectin fibrils, Kallikrein-related peptidase

**Short title:** KLK12 –mediated fibronectin matrix remodeling.

Correspondence should be addressed to N.HV: CEPR INSERM U1100, Faculté de Médecine, 10 Boulevard Tonnellé, F-37032 Tours cedex, France. Tel.: 33-247366237; Fax:

33-247366046; E-mail: nathalie.vourch@med.univ-tours.fr


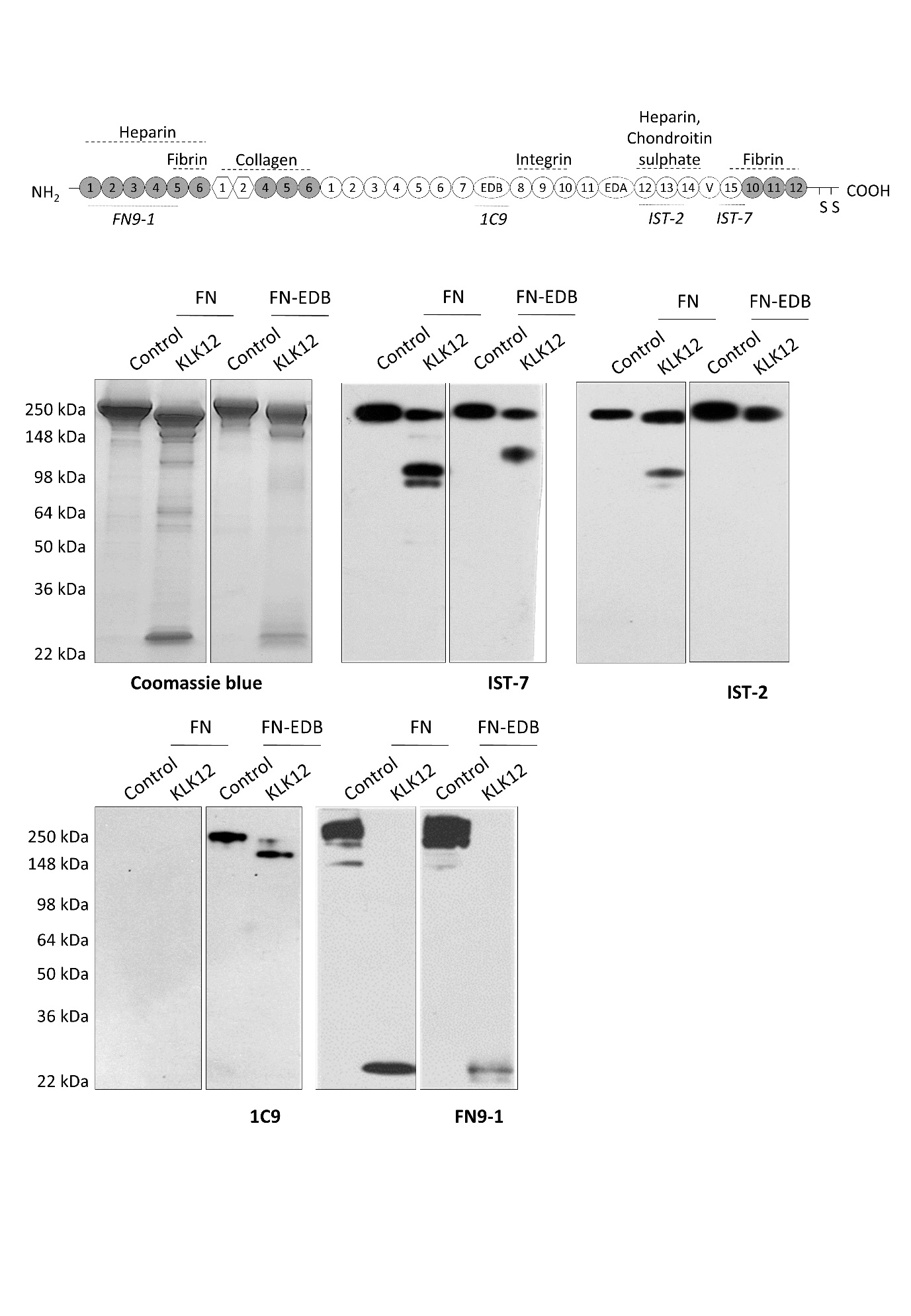


**Supplementary Figure 1: Western blotting detection of fibronectin proteolytic fragments generated by KLK12.**

**A)** A schematic diagram of FN’s domain structure. Intra- and intermolecular interactions are indicated above the diagram. Domains recognized by antibodies used in this study are indicated below the diagram. **B)** Western blots were performed on FN proteolytic fragments generated by KLK12 (as in Figure 2A), using a set of four different antibodies directed against specific FN domains.
